# Modifications of Dental Implant Surfaces at the Micro- and Nano-Level for Enhanced Osseointegration

**DOI:** 10.3390/ma13010089

**Published:** 2019-12-23

**Authors:** In-Sung Luke Yeo

**Affiliations:** Department of Prosthodontics, School of Dentistry and Dental Research Institute, Seoul National University, Seoul 03080, Korea; pros53@snu.ac.kr; Tel.: +82-2-2072-2662

**Keywords:** surface modification, osseointegration, SLA, TiO_2_ nanotube, fluoride, photofunctionalization

## Abstract

This review paper describes several recent modification methods for biocompatible titanium dental implant surfaces. The micro-roughened surfaces reviewed in the literature are sandblasted, large-grit, acid-etched, and anodically oxidized. These globally-used surfaces have been clinically investigated, showing survival rates higher than 95%. In the past, dental clinicians believed that eukaryotic cells for osteogenesis did not recognize the changes of the nanostructures of dental implant surfaces. However, research findings have recently shown that osteogenic cells respond to chemical and morphological changes at a nanoscale on the surfaces, including titanium dioxide nanotube arrangements, functional peptide coatings, fluoride treatments, calcium–phosphorus applications, and ultraviolet photofunctionalization. Some of the nano-level modifications have not yet been clinically evaluated. However, these modified dental implant surfaces at the nanoscale have shown excellent in vitro and in vivo results, and thus promising potential future clinical use.

## 1. Introduction

The surface quality of titanium (Ti) dental implants, which replace missing teeth, is one of the keys to the long-term clinical success of implants in a patient’s mouth [1]. The bone response to the Ti implant surface depends on its surface characteristics: Contact (bone formation on the implant surface towards the bone) and distance osteogenesis occur around micro-roughened Ti surfaces while only distance osteogenesis (bone formation from the old bone toward the implant surface) appear around turned Ti [2]. Although contact osteogenesis seems to require other factors to be triggered, modification of the implant surface is very important to accelerate osseointegration [3].

Ti is known to be stable in biologic responses and not to trigger a foreign body reaction when inserted into the human body [4,5]. Therefore, osseointegration was originally defined as the direct contact between a loaded implant surface and bone at the microscopic level of resolution [1]. Recently, this term has been interpreted from a new point of view: Osseointegration is essentially a demarcation response to a foreign body of Ti when the Ti implant is immobile in bone [6]. This demarcation is immune-driven and is classified as a type IV hypersensitivity [7]. Based on the original definition, the modification of a Ti implant surface implies that the surface would be more biocompatible, thereby increasing the bioaffinity of the hard tissue and accelerating the bone response to the surface. The new standpoint on osseointegration suggests that the modified Ti surface would be recognized more sensitively by the hard tissue, which would isolate this foreign body with a faster and stronger accumulation of bone substances. Thus, the nature of osseointegration is under investigation at present [8]. The detection of the actual bond between the bone and implant surfaces could support the bioaffinitive nature of bone response to the surfaces [9,10]. Only friction and physical contact would exist at the interface if the bony demarcation hypothesis is correct.

To date, implant surfaces have been modified in various ways under the bioaffinity concept for osseointegration. Conventionally, the topography of the surface has been changed at the micro-level (1–10 μm). At present, some chemical features and nanotechnologies have been added to the surfaces. This review introduces several recent advancements of biocompatible implant surfaces with a few representative micro-roughened modified surfaces. Since most implant surfaces used in the global market have been made of commercially pure Ti (cp-Ti), especially grade 4 cp-Ti, this review is based on the modification of a grade 4 cp-Ti surface. 

## 2. Micro-Roughened Modification

### 2.1. Sandblasted, Large-Grit, Acid-Etched (SLA) Surface

**Figure 1:**
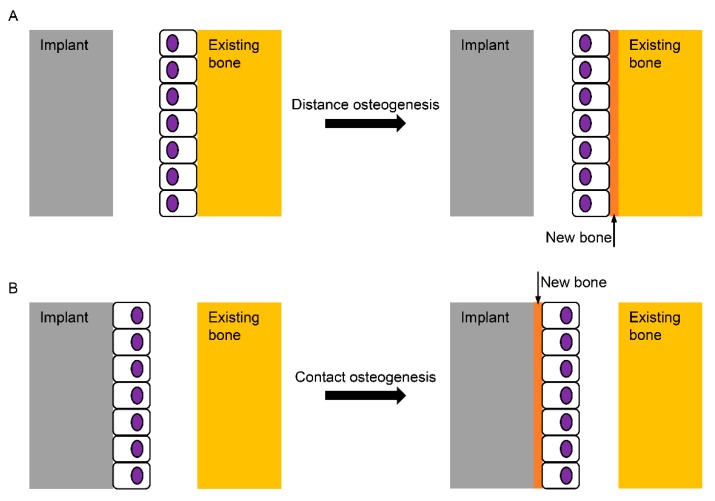
Schematic diagram for the healing mechanisms of the bone surrounding an implant. (**A**) In distance osteogenesis, the direction of bone formation is from the existing bone to the implant; (**B**) in contact osteogenesis, however, the direction is opposite, from the implant to the existing bone, which is known not to occur on the turned Ti (Titanium) surface without any modification.

The computer numerical controlled milling of cp-Ti manufactures screw-shaped endosseous dental implants. The surface machined by this milling procedure, which is now called a turned Ti surface, shows many parallel grooves in scanning electron microscopy (SEM). The turned surface experiences no modification process, which has frequently served as a control to evaluate the biocompatibility of modified surfaces. When an implant is inserted into the bone and the implant surface becomes juxtaposed to the bone, bone healing (or osseointegration) on the surface is known to be fulfilled by two mechanisms: distance and contact osteogenesis [2,11]. In distance osteogenesis, new bone starts to be formed on the surfaces of bone. The direction of bone growth is from the bone towards the implant surface (Figure 1A). In contact osteogenesis, or de novo bone formation, new bone formation begins on the implant surface. The direction of bone growth is from the implant towards the bone, opposite to that for distance osteogenesis (Figure 1B). When an endosseous implant with a turned surface is placed into the jawbone, only distance osteogenesis occurs, which implies that more time is needed for sufficient osseointegration to withstand masticatory forces [2,12]. The necessity of reduction in the patient’s edentulous period has led the modification of an implant surface to accelerate bone healing.

The traditional approach to the surface modification of a Ti implant has been roughening at the micro-level. One of the most successful surfaces in clinical dentistry is the sandblasted, large-grit, and acid-etched (or SLA) surface. An SLA Ti surface is made by sandblasting the turned Ti surface with large-grit particles, the sizes of which range from 250 μm to 500 μm in general, and by acid-etching the blasted surface. The acids for etching are usually strong acids including hydrochloric, sulfuric, and nitric acids. SEM shows topographically changed irregularities on the SLA surface, with large dips, small micropits, sharp edges, and pointed tips. Sa, one of the surface parameters defined as the arithmetic mean height of the surface, is approximately 1.5 μm to 2 μm. Osteogenic cells migrate to the roughened Ti surface through the fibrin clot that is formed at the peri-implant site after bone drilling for implant insertion, and these cells appear to recognize the irregularities of the SLA surface as lacunae to be filled with bone materials [2,13]. Contact osteogenesis occurs as the osteogenic cells secrete a bone matrix. The occurrence of both contact and distance osteogenesis accelerates the osseointegration on the SLA surface compared to the turned surface.

The Ti surface of a dental implant is originally hydrophobic [14]. Water (H_2_O) is considered to have initial contact with the implant surface when the implant is inserted into the bone [15]. Therefore, there have been attempts to add hydrophilicity to an SLA surface, since hydrophilicity is expected to help accelerate the bone healing process [14,16]. A dental implant with a hydrophilic SLA surface, commercially called SLActive (Institute Straumann AG, Basel, Switzerland), is made with a water rinse of the original SLA implant in a nitrogen chamber and a packaging technique of storing the implant in an isotonic sodium chloride solution with no atmospheric contact, and this hydrophilic implant is being clinically used in the global market [17].

Regardless of whether an SLA surface is hydrophobic or hydrophilic, this dental implant surface has shown excellent long-term clinical results [18,19,20,21,22]. A previous 10-year retrospective study investigating more than 500 SLA Ti implants concluded that both the survival and success rates were 97% or higher [18]. The 10-year survival rate of SLA Ti implants was reported to be higher than 95%, even in periodontally compromised patients, although strict periodontal interventions were applied to these patients [20]. Similar results were found in 10-year prospective studies investigating the survival rates of dental implants with SLA surfaces [19,21,22]. This modified surface, roughened at the micro-scale, is one of the dental implant surfaces that has been most frequently tested in clinics for the longest period. 

### 2.2. Anodic Oxidation

The genuine biocompatible surface on the Ti dental implant is Ti oxide (TiO_2_), not Ti itself, which is spontaneously formed when the Ti surface is exposed to the atmosphere. However, this Ti oxide layer is very thin (a few nm in thickness) and is imperfect with defects [23]. Also, chemically unstable Ti^3+^ and Ti^2+^ are known to exist in the oxide layer [24]. Therefore, there have been several techniques developed to thicken and stabilize the Ti oxide layer, which is considered to increase the biocompatibility of the surface [25,26,27]. When Ti becomes the anode under an electric potential in an electrochemical cell, Ti is oxidized to be Ti^4+^, and the TiO_2_ layer is able to be thickened and roughened [15]. Topographically, the oxidized Ti surface for a dental implant has many volcano-like micropores with various sizes, which are observed in SEM. The surface characteristics of the anodized Ti surface depend on the applied potential, surface treatment time, concentrations, and types of electrolytes [15,27]. The arithmetic mean height of this surface, or Sa, is evaluated to be approximately 1 to 1.5 μm for dental use [28,29,30,31].

Osteogenic cells appear to recognize the topography of a dental implant surface although we do not yet know which surface topography is more proper in bone healing, or if the irregularities of the SLA surface are more effective for the osteogenic cell response than the microporous structure of the anodized surface [32]. To date, no in vivo model has found any significant differences in bone responses to the microtopographies of Ti dental implant surfaces [33,34]. What is definitely known about implant surface topography is that the cp-Ti surfaces topographically modified at the microscale accelerate osseointegration more than the turned surface, and these modified surfaces show superior results to the turned surface during in vitro, in vivo, and clinical studies.

The anodically oxidized Ti surface has shown superior results to the turned surface in various in vitro tests and in vivo histomorphometry [31,34,35,36]. A previous meta-analytic study reported lower failure rates of the oxidized Ti implants than those of the turned implants from the included 38 clinical investigations [37]. A prior retrospective and a 10-year prospective study concluded that that success rates were higher than 95% for the TiUnite surface (Brånemark System, Nobel Biocare, Göteborg, Sweden), which is a trade name for the oxidized Ti surface [38,39]. However, a recent 20-year randomized controlled clinical trial notably reported a similar marginal bone loss between micro-roughened and turned Ti implants [40]. This clinical study used an identical implant design with an implant-abutment connection structure and internal friction connection [40]. Identifying which of the two factors (surface characteristics and implant design) is a major contributor to the long-term clinical success of dental implants needs to be thoroughly investigated, although higher success or survival rates have been steadily published for Ti dental implants with modified surfaces at the micro-scale, compared to the turned implant [19,41,42].

## 3. Molecular Modification

### 3.1. TiO_2_ Nanotube

**Figure 2:**
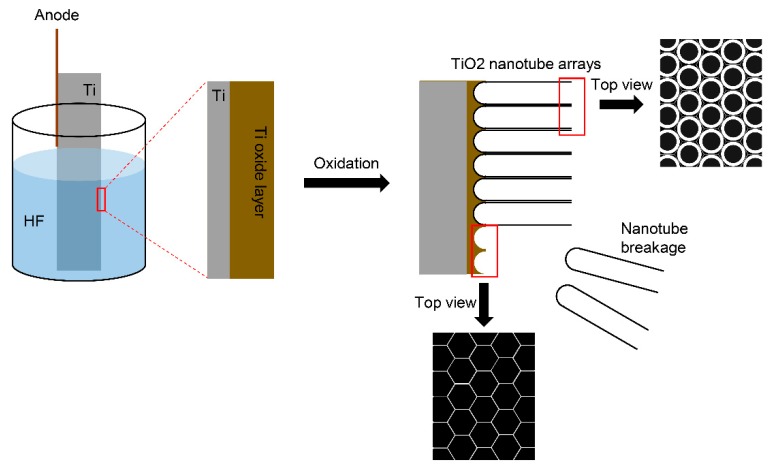
Schematic diagram showing the formation of TiO_2_ nanotube arrays. In the electrolyte solution containing hydrogen fluoride (HF), regular tube structures are formed on the Ti surface of the anode at a nanoscale. When the structures are viewed on top, the circular forms of the tubules are found via scanning electron microscopy. The binding between the nanotube arrays and Ti surface is generally weak, and breakdown is frequent at the interface. The morphology underneath the tubes is hexagonal.

Anodic oxidation is extended to the modification of a Ti dental implant at the nanoscale (1–100 nm). The electric current of the electrochemical cell, temperature, the pH values of electrolyte solutions, the electrolytes, oxidation voltage, and oxidation time affect the nanotopographies of the Ti surface [43,44]. In an electrochemical cell composed of Ti at the anode and platinum (or Ti) at the cathode, the TiO_2_ layer is normally formed on the Ti implant surface of the anode [43]. In an appropriate fluoride-based electrolyte, the nano-morphology of the TiO_2_ layer is changed, and the aligned TiO_2_ nanotube layer is developed (Figure 2) [43].

In the past, implant surface nanostructures were reported to have no effect on cell responses and bone responses to dental implant surfaces and were thought to depend on the microtopographies of the surfaces [45,46]. Optimal micro-roughness is known at present to be 1.5 μm in Sa and approximately 4 μm in diameter of the surface irregularities [30,47]. However, a previous review article noted that the microtopographies of the dental implant surfaces have a limited influence on the initial responses of the in vivo hard tissue environment [48]. Presently, the nanotopographical features of Ti implant surfaces have been known to be contributors to the initial biologic responses of the hard tissue, including osteoblast activities and osteoclast reactions [44,49].

This modified surface with TiO_2_ nanotube arrays is highly biocompatible [44,50,51]. Both osteoblasts and osteoclasts showed maximal cellular responses to Ti surfaces with TiO_2_ nanotubes that were 15 nm in diameter [52]. Interestingly, smaller TiO_2_ nanotubes, which were approximately 30 nm in diameter, were more effective in the adhesion and growth of mesenchymal stem cells than larger TiO_2_ nanotubes that ranged from 70 nm to 100 nm, while the latter TiO_2_ nanotubes were more inductive in the differentiation into osteoblast-like cells, although there is contrary to previous studies [52,53]. The modified TiO_2_ nanotubular surface showed excellent bone-to-implant contact in the osteoporotic bone in an in vivo study using ovariectomized rats [54].

Another characteristic of this nano-modified surface is a drug delivery effect [55,56,57,58]. Drug release from TiO_2_ nanotubes is associated with the dimensions of TiO_2_ nanotube arrays regardless of the direct release or indirect discharge by nanocarriers [59]. The diameter and length of TiO_2_ nanotubes generally increase as the voltage and duration of the oxidation process increase, and the drug release has been found to be effective when the diameter is larger than approximately 100 nm [56,59,60]. A combination of this nano-modified TiO_2_ surface and carrier molecules, including micelles, is being actively investigated for drug delivery at a constant rate, unrelated to the drug concentration and release period [57,58,60].

The nanotopography of the TiO_2_ nanotubular surface has antibacterial properties alongside delivering antibiotic drugs [61]. Streptococcus mutans, which are associated with the initial formation of biofilm in the oral cavity, were reported to adhere to the TiO_2_ nanotube arrays less than to a micro-roughened SLA surface [62]. The hydrophilic properties of TiO_2_ nanotubes seems to hinder bacterial adhesion to the nanotubular surface [62]. However, it is notable that many studies have described the wettability of the TiO_2_ nanotube arrays, showing conflicting results in cellular and bacterial responses to the nanotubular surface [61,63,64]. Although the hydrophilicity of the TiO_2_ nanotube arrays is adjustable, some studies reported that the reduction of bacterial adhesion was due to the hydrophilic properties of the surface, whereas other studies described that such a result was due to the hydrophobic properties [61,63,64]. Further investigation is required to determine the mechanism of bacterial and cellular responses to the wettability of Ti surfaces.

Despite that the modified surface with TiO_2_ nanotube arrays has very useful advantages (e.g., high biocompatibility, the capability of drug delivery, and antibacterial properties), this surface has been neither applied nor tested clinically. The mechanical strength between the TiO_2_ nanotubes and the base Ti surface is too weak for this surface to be applied to a dental implant [43]. Recently, the hexagonal nano-structure of the base Ti surface was evaluated to be adequate for biologic application when the TiO_2_ nanotube arrays are removed from the base surface in order to prevent the delamination of the TiO_2_ nanotube coating in an in vivo environment (Figure 2) [44]. The aligned TiO_2_ nanotube-layered surface has great potential in biologic and clinical applications [55,56,57,65]. However, it is necessary to overcome this delamination problem before this TiO_2_ nanotubular surface is clinically used in the field of dental implantology.

### 3.2. Functional Peptides

Water and ions have first contact with the implant surface when the bone is drilled for implant insertion and a screw-shaped endosseous dental implant is placed into the bone. Then, the plasma proteins adhere to the surface through ionic bridges (like a calcium ion linkage), and the fibrin clot is formed. During hemostasis, extracellular matrix (ECM) proteins gradually replace the plasma proteins [15]. The adhesion proteins, including fibronectin and vitronectin (which are also ECM proteins), are recognized by the transmembrane proteins of osteogenic cells like integrins. Through binding of the transmembrane proteins to the osteogenic cells, the cells interact with ECM, which controls the cellular activities for bone healing [66]. Therefore, the bone healing process starts from the adhesion of the osteogenic cells to surfaces, and these adhesion proteins can play a role in accelerating osseointegration into dental implants when the proteins are applied to the implant surfaces. Core amino acid sequences, which are extracted from the original adhesion proteins and still have binding activities to the transmembrane receptors, are very useful in rapid bone healing when the core sequences are treated on the implant surfaces. These core functional peptides are considered to be more promising candidates for implant surface treatment than the original proteins because of the lower antigenicity and simpler adjustability of the peptides [67].

A functional peptide derived from the fibronectin, arginyl-glycyl-aspartic acid sequence, revealed improved histomorphometric results when this peptide was coated on a Ti dental implant surface and when this peptide-treated surface was compared to the uncoated surface [68]. Two functional amino acid sequences derived from another adhesion protein, laminin, showed excellent results as accelerating modifiers for Ti implant surfaces for osseointegration [35,67]. These functional peptides based on the adhesion of osteogenic cells seem to surpass the effects of the microtopographical features of the underlying Ti implant surfaces in bone healing, although further studies are definitely needed [35,69]. The mechanism behind the superior bone cell responses has been tried to be explained, based on the hypothesized tunable allosteric control of the receptor proteins [67,70]. A recent investigation evaluating a functional peptide from vitronectin found a Janus effect of this peptide for bone formation, activating osteoblasts and inhibiting osteoclasts, that is, controlling the osteoporotic environment locally to be favorable for osseointegration [71].

Cytokines, particularly growth factors, are another class of bioactive proteins. Bone morphogenetic proteins (BMPs) are available for bone healing in the field of dental implantology. Human recombinant BMP-2 (rhBMP-2) is used in the global market for bone regeneration. BMP-2 is known to have a direct effect on osteogenic cells to promote bone formation with various interactions between this protein and other bioactive molecules, including osteogenic genes [72,73]. However, these growth factors have many problems to be solved before clinical application to Ti dental implant surfaces. BMP-2 has complicated biologic effects depending on its concentrations and surroundings; osteogenesis, adipogenesis, and chodrogenesis, but osteolysis also occurs [72,74,75]. The rhBMP-2-treated Ti surface was reported to make bone healing around a dental implant faster in an in vivo model [76,77]. However, it is recognizable that growth factors are usually active in free forms, not in bound forms. Therefore, these molecules are ineffective or, if any, limitedly active when the factors are bound or attached to implant surfaces [78]. The cell transmembrane proteins that recognize these growth factors are disengaged in the attachment of the cells [78]. Because of the multiple enigmatic effects of these growth factors on living tissues and the growth factor receptors’ lack of involvement in cell adhesion, growth factor-treated implant surfaces have not been used clinically until now.

Although these bioactive molecules, including the adhesion molecules and growth factors, have the potential to be applied to dental implants for accelerated osseointegration, the Ti dental implants on which these molecules are coated have not been clinically tested; there have been no published clinical trials to report the results of such implants. The functional peptides from the adhesion molecules are to be clinically tried and applied in dental implantology in the near future due to the simplicity in their biologic effects and their low probability of side effects. For growth factors, it seems to be necessary to find core amino acid sequences from growth factors to increase the clinical applicability of these factors. Before these derived peptides are clinically tried, further studies are required on release strategies for the molecules from the implant surfaces and on the biologic activities of the core peptides.

### 3.3. Fluoride Treatment (Cathodic Reduction)

When a Ti implant is a cathode in the hydrofluoric acid solution of an electrochemical cell, a fluoride ion gives an electron to the cathode, where the reduction of a Ti ion occurs. As a result, a trace amount of fluoride ions adheres to the Ti implant surface when the concentration of hydrogen fluoride is low in the solution. This trace amount of fluoride ions is known to primarily affect osteoprogenitor cells and undifferentiated osteoblasts to enhance bone formation, rather than highly differentiated osteoblasts [79,80]. Furthermore, fluoride is helpful for bone mineralization because of its properties that are attractive for calcium [78]. However, fluoride ions are thought to become cytotoxic as the number of ions increases on the Ti implant surface.

Clinically, a modified surface is used as a dental implant surface (Osseospeed, Astra Tech, Dentsply, Waltham, MA, USA), which is fluoride-treated after the grade 4 cp-Ti is sandblasted with TiO_2_ particles. This fluoride-modified surface has a very low amount of fluoride, which is difficult to find by energy dispersive spectroscopy, while x-ray photoelectron spectroscopy is able to detect this trace amount [81,82]. The average mean height of this marketed surface has been investigated to be approximately 1.5 μm [30,82]. The fluoride-treated Ti surface has shown stronger binding between the bone and this surface than the control Ti surface without fluoride-treatment [83,84]. However, finding any significant differences in the histomorphometric results has been very rare when the fluoride-treated dental implants have been compared in vivo to other modified implants, including SLA implants, while some previous studies have been found to show more favorable results in bone responses to the fluoride-treated surface than those to its predecessor with no application of fluoride [78,82,85,86].

Dental implants with a fluoride-treated surface have exhibited high rates of success and survival rates in clinical trials. These fluoride-treated implants have supported prosthodontic restorations in edentulous mandibles with a 100% survival rate for ten years [87]. Regardless of the maxilla or the mandible, high survival rates of over 95% have been reported for the surface-modified dental implants in the prospective clinical studies, the observation periods of which are longer than 5 years [87,88,89]. It is notable and very interesting that these previous clinical studies have consistently reported the vertical loss of bone surrounding the implants of less than 0.5–1 mm, which is interpreted as almost no change of the bone level [40,87,89]. Importantly, these clinical studies used fluoride-treated dental implants with the same implant macro-design, including an identical thread shape and internal friction implant–abutment connection, so care must be taken when interpreting data in comparison studies of the biologic responses between the dental implant systems [82]. It remains uncertain which factor (surface chemistry in fluoride treatment, surface topography, implant–abutment connection tectonics) is a major contributor to the biologic responses in humans to this marketed fluoride-treated dental implant.

### 3.4. Hydroxyapatite and Other Calcium–Phosphorus Compounds

This idea of hydroxyapatite (HA) coating on a Ti dental implant surface is based on the fact that the main component of bone is HA. HA (Ca_10_(PO_4_)_6_(OH)_2_) is still the most commonly-utilized coating material for Ti dental implant surfaces [90]. HA and other calcium-phosphorus coating materials are basically osteoconductive to the surrounding bone. The biologic features of these materials, such as their biodegradation properties and foreign body reactions, seem to depend on calcium/phosphorus ratios, crystallinities, and coating thicknesses [31,90,91,92,93]. Plasma spraying (a conventional atmospheric plasma-spray method) is one of the most widely used methods to coat HA on a Ti implant surface [90]. HA particles that are contained and heated in a plasma flame whose temperature is approximately 15,000 to 20,000 Kelvin are sprayed on the Ti surface, resulting in a HA coating layer that is 50–100 μm in thickness [94]. The spray parameters, including the flame combination and spraying flow rate, affect the chemical and physical features of the HA coating [92].

The HA coating is biocompatible with the hard tissue, showing direct contact with bone and the attachment of osteoblasts on the coating surface [95,96]. Many studies have reported enhanced bone apposition and the prevention of metal-ion release into the bone from metal implants with an HA coated surface [97,98,99,100,101]. However, the HA coating layer has some critical issues to be addressed. Like the TiO_2_ nanotube arrays, the delamination of the coating layer from the Ti dental implant surface is one of the problems (adhesive failure) [92]. Delaminated or worn HA particles hinder bone healing and provoke inflammation around the implant inserted into the bone [92,102]. The thick coating layer is able to make a breakage inside the layer, especially at the implant in a load-bearing area (cohesive failure) [92]. Recently, a thin calcium–phosphorus coating layer has been achieved and investigated using various coating techniques [76,93,101,103]. Compared to the plasma sprayed HA coating, however, the other calcium–phosphorus coatings are considered to lack long term clinical results [104,105].

The five-year clinical success rate of the HA coated implant has been evaluated to be approximately 95% [106]. However, this success rate has dropped markedly to below 80% after 10 years of implant placement [106,107,108]. Such a low success rate may result from the above-mentioned problems of the HA coating layer. It is notable, however, that these clinical evaluations resulted from the data of cylindrical implants [107,108]. A previous study using HA coated screw-shaped implants (MicroVent, Zimmer Dental, Carlsbad, CA, USA) reported that the long-term clinical success rate (> 10 years) was higher than 90% [109]. Nevertheless, clinical trials are certainly necessary to evaluate the calcium–phosphorus coating more precisely.

### 3.5. Photofunctionalization

In 1997, it was determined that the wettability of the TiO_2_ surface is increased by ultraviolet (UV) radiation [110]. Originally, the UV-induced TiO_2_ surface is amphiphilic—both hydrophilic and oleophilic [110]. However, the enhanced biologic effect of this surface is considered to be caused by the hydrophilic properties. Such hydrophilicity and elimination of hydrocarbon contamination on the TiO_2_ surface are known to be the mechanisms behind further activated bone responses to a dental implant in UV-mediated photofunctionalization. The hard tissue affinity drops for an aged Ti surface that has been stored for longer than two weeks [111]. UV irradiation on the Ti implant surface appears to make the Ti surface reactivate, as the implant is freshly made.

UV radiation is subcategorized into three types according to its wavelengths and dermal biologic reactions to the electromagnetic waves: UVA, UVB, and UVC [112]. The wavelengths of UVA range from 320 to 400 nm, and those of UVC range from 200 to 280 nm [113]. Both UVA and UVC contribute to increasing the hydrophilicity of the Ti surface. However, considering the fact that some reports show the promoted osteogenic activities on hydrophobic surfaces, the removal of carbon from the Ti surface, which is caused by UVC, is likely a fundamental mechanism behind excellent osseointegration [114,115,116,117]. Strictly, neither UVA nor UVC appears to make a topographic change at the nano-scale on the Ti surface [115,117]. Friction force microscopy shows a nano-scale modification that UV irradiation may produce by converting Ti^4+^ to Ti^3+^ [110]. UV treatment on the Ti surface enhances the adsorption of proteins, such as albumin and fibronectin, which are plasma proteins in the human body [118]. UV-photofunctionalized implant surfaces show improved osteogenic cell attachment, spreading, and proliferation [117]. The antibacterial effects are described for the UV activation of the Ti surface [112]. Faster bone responses to UV-treated Ti surfaces are reported in various in vivo studies, some of which show almost 100% bone-to-implant contact [117,118,119,120].

A previous clinical study showed that the stability of implants inserted into the patients’ jaw bones increased more rapidly when the implants were UV-photofunctionalized [121]. The retrospective clinical studies concluded that UV-mediated photofunctionalization reduced early implant failure, and the success rate of the photofunctionalized implants was 97.6% during the functional loading period of approximately 2.5 years [122,123]. No prospective long-term clinical study (published in English) evaluating UV-mediated photofunctionalization has yet been found in the field of implant dentistry. However, a prospective clinical evaluation of UV-treated implants over more than 5 years is expected to be published shortly.

### 3.6. Laser Ablation

For laser ablation, an implant whose collar, or neck area, was treated by laser micromachining to generate nano-channels is used (Laser-Lok, BioHorizons, Birmingham, AL, USA) [124,125]. Laser ablation is also able to produce micro-scale patterns by controlling laser processing parameters [126]. This approach was intended to promote not only fast osseointegration, but also connective tissue attachment [124,127]. The connective tissue fiber direction in the soft tissue attachment is known to be perpendicular to the laser-microtextured Ti implant surface, which is characteristically different from the general orientation of the fibers parallel to implant surfaces [127,128]

This marketed laser-modified surface (Laser-Lok) showed significantly improved bone-to-implant contact in a previous in vivo study, compared to a turned Ti surface [129]. The survival rate was evaluated to be 95.6% in a two-year retrospective multicenter study and to be 94% in another 5-year retrospective controlled study [127,130]. Recently, the prospective three-year results of a randomized clinical trial were reported for single implant-supported restorations with the laser-modified Ti implant surface, where the survival rate was estimated to be 96.1% [131]. Both the hard and soft tissue responses to the laser-modified Ti surface appear to be favorable [127,130,131,132]. However, long-term prospective clinical results of laser micromachining are still needed.

## 4. Concluding Remarks

When the bone is prepared for implant placement, surgical trauma provokes bleeding and hemostasis. Moreover, this surgical trauma activates the growth and differentiation factors released from the bone debris and matrix [15]. Surface modification of the Ti dental implant focuses on improving such initial biologic responses to the implant surface. Researchers and dental clinicians anticipate the best performance of the implant surfaces during these initial events and more readily establish these events by changing the physical and chemical properties of the surfaces, thereby boosting the speed and strengthening the quality of the healing process [133]. However, as the long-term clinical studies show, implant-supported prostheses have been used for a long time in patients’ mouths. Therefore, the modified surfaces also need to harmonize with the bone remodeling process, which has not yet been investigated. This paper reviews several modified surfaces of dental implants that are widely used in the global market or are highly possible to be clinically used. All these reviewed surfaces are targeted to accelerate early bone responses. The late responses of the hard tissue to the surfaces, including bone remodeling, need to be investigated. Moreover, long-term clinical trials are still required for these implant surfaces.
